# A unified phylogeny-based nomenclature for histone variants

**DOI:** 10.1186/1756-8935-5-7

**Published:** 2012-06-21

**Authors:** Paul B Talbert, Kami Ahmad, Geneviève Almouzni, Juan Ausió, Frederic Berger, Prem L Bhalla, William M Bonner, W Zacheus Cande, Brian P Chadwick, Simon W L Chan, George A M Cross, Liwang Cui, Stefan I Dimitrov, Detlef Doenecke, José M Eirin-López, Martin A Gorovsky, Sandra B Hake, Barbara A Hamkalo, Sarah Holec, Steven E Jacobsen, Kinga Kamieniarz, Saadi Khochbin, Andreas G Ladurner, David Landsman, John A Latham, Benjamin Loppin, Harmit S Malik, William F Marzluff, John R Pehrson, Jan Postberg, Robert Schneider, Mohan B Singh, M Mitchell Smith, Eric Thompson, Maria-Elena Torres-Padilla, David John Tremethick, Bryan M Turner, Jakob Harm Waterborg, Heike Wollmann, Ramesh Yelagandula, Bing Zhu, Steven Henikoff

**Affiliations:** 1 Howard Hughes Medical Institute, Basic Sciences Division, Fred Hutchinson Cancer Research Center, Seattle, WA, 98109, USA; 2 Department of BCMP, Harvard Medical School, Boston, MA, 02115, USA; 3 CNRS, UMR 218, Institut Curie, Centre de Recherche, Paris, F-75248 cx 05, France; 4 Department of Biochemistry and Microbiology, University of Victoria, Victoria, BC, V8W 3P6, Canada; 5 Temasek Lifesciences Laboratory, 1 Research Link, National University of Singapore, Singapore, 117604, Singapore; 6 Plant Molecular Biology and Biotechnology Group, Melbourne School of Land and Environment, The University of Melbourne, Parkville, VIC, 3010, Australia; 7 Laboratory of Molecular Pharmacology, CCR, NCI, NIH, Bethesda, MD, 20892, USA; 8 Department of Molecular and Cell Biology, University of California, Berkeley, CA, 94720-3200, USA; 9 Department of Biological Science, Florida State University, Tallahassee, FL, 32306-4295, USA; 10 Department of Plant Biology, UC Davis / HHMI, Davis, CA, 95616, USA; 11 Laboratory of Molecular Parasitology, The Rockefeller University, New York, NY, 10065, USA; 12 Department of Entomology, Pennsylvania State University, University Park, PA, 16802, USA; 13 Laboratoire de Biologie Moléculaire et Cellulaire de la Différenciation, Institut Albert Bonniot, INSERM/UJF U821, Grenoble, France; 14 Department of Biochemistry, University of Goettingen, Goettingen, D-37073, Germany; 15 Department of Cellular and Molecular Biology, University of A Coruna, A Coruna, E15071, Spain; 16 Department of Biology, University of Rochester, Rochester, NY 14627, USA; 17 Center for Integrated Protein Science Munich at the Adolf-Butenandt Institute, Department for Molecular Biology, Ludwig-Maximilians-University Munich, Munich, 80336, Germany; 18 Department of Molecular Biology and Biochemistry, University of CA, Irvine, CA, 92697, USA; 19 Howard Hughes Medical Institute, Department of Molecular Cellular and Developmental Biology, University of California, Los Angeles, CA, 9009, USA; 20 Max Planck Institute for Immunbiology and Epigenetics, Freiburg, 79108, Germany; 21 INSERM, U823; Université Joseph Fourier - Grenoble 1, La Tronche, F-38706, France; 22 Department of Physiological Chemistry, Butenandt Institute and Biomedical Center, Ludwig Maximilians University of Munich, Munich, 81377, Germany; 23 National Center for Biotechnology Information, National institutes of Health, Bethesda, MD, 20894, USA; 24 CGphiMC - CNRS UMR5534, Claude Bernard University Lyon1 - University of Lyon, Villeurbanne, 69622 Cedex, France; 25 Program in Molecular Biology and Biotechnology, University of North Carolina, Chapel Hill, NC, 27599, USA; 26 Department of Animal Biology, University of Pennsylvania, Philadelphia, PA, 19104-6046, USA; 27 HELIOS Medical Centre Wuppertal, Paediatrics Centre, Witten/Herdecke University, Wuppertal, D-42283, Germany; 28 Institut de Genetique et Biologie Moleculaire et Cellulaire, Illkirch, 67404, France; 29 Department of Microbiology, Immunology, and Cancer Biology, University of Virginia, Charlottesville, VA, 22908, USA; 30 Sars International Center for Marine Molecular Biology and Department of Biology, University of Bergen, Bergen, N-5008, Norway; 31 Stem Cells and Developmental Biology, Institut de Génétique et de Biologie Moléculaire et Cellulaire, CNRS/INSERM U964, Universite de Strasbourg, Illkirch, CU de Strasbourg, F-67404, France; 32 The John Curtin School of Medical Research, Genome Biology Department, The Australian National University, Canberra, ACT 2601, Australia; 33 College of Medical and Dental Sciences, University of Birmingham, Birmingham, B15 2TT, UK; 34 Cell Biology and Biophysics, School of Biological Sciences, University of Missouri-Kansas City, Kansas City, MO, 64110, USA; 35 Chromatin Lab, National Institute of Biological Sciences, Beijing, Beijing, 102206, China

## Abstract

Histone variants are non-allelic protein isoforms that play key roles in diversifying chromatin structure. The known number of such variants has greatly increased in recent years, but the lack of naming conventions for them has led to a variety of naming styles, multiple synonyms and misleading homographs that obscure variant relationships and complicate database searches. We propose here a unified nomenclature for variants of all five classes of histones that uses consistent but flexible naming conventions to produce names that are informative and readily searchable. The nomenclature builds on historical usage and incorporates phylogenetic relationships, which are strong predictors of structure and function. A key feature is the consistent use of punctuation to represent phylogenetic divergence, making explicit the relationships among variant subtypes that have previously been implicit or unclear. We recommend that by default new histone variants be named with organism-specific paralog-number suffixes that lack phylogenetic implication, while letter suffixes be reserved for structurally distinct clades of variants. For clarity and searchability, we encourage the use of descriptors that are separate from the phylogeny-based variant name to indicate developmental and other properties of variants that may be independent of structure.

## 

Histones, the basic proteins that wrap DNA into nucleosomes in eukaryotes, are commonly encoded by multigene families. Histones fall into five protein families, the core histones H2A, H2B, H3 and H4, and the linker histone family H1. A nucleosome core particle is made by assembling two proteins from each of the core histone families together with DNA. Linker DNA between core particles may be bound by a member of the H1 family. The individual paralogous (non-allelic) genes of a histone family may encode identical proteins, or they may encode related but distinct protein isoforms, commonly referred to as “histone variants”. Though histone variants have been known almost from the beginning of histone research, we are still discovering the diversity of their roles and functions. Histone variants play critical roles in such diverse processes as transcription, chromosome segregation, DNA repair and recombination, chromatin remodeling, ADP-ribosylation, germline-specific DNA packaging and activation, and even extra-nuclear acrosomal function. Some variants of H2A and H3 have well-studied, specialized functions. Variants of H1 and H2B are common, but much less is known of their functional specialization. H4 variants are few.

The diversity of core histones has led to confusion in naming since their discovery. The current names - H2A, H2B, H3 and H4 - for the ‘canonical’ histones were agreed upon at the Ciba Foundation Symposium in 1975 to simplify and standardize competing names for these proteins based on different methods of extraction [1]. At that time, the first core histone variants of H2A, H2B and H3 had already been described from *Drosophila*[2], from the sea urchin *Parechinus angulosus*[3] and from calf thymus [4,5], respectively. Since then, the numerous variants that have been described from these four basic protein families have been named in a variety of styles, using various combinations of numbers, letters and punctuation. The lack of systematized names can lead to confusion of similar names and incorrect attributions of orthology or common function. For instance, H2Bv from *Plasmodium* can be confused with H2BV from *Trypanosoma*, though the two variants are not closely related. Conversely, the same variant can go by different names in different organisms. A PubMed search for H2A.Z or H2A.Z* gets 269 papers on this variant, but misses 126 that use the names D2, H2Az, Htz1, H2A.F or H2A.F/Z, hv1, H2Av or H2AvD.

At a time when genome sequencing has become routine, the discovery of new variants is common. Indeed, lineage-specific expansions of paralogs confound simple orthology and produce a wealth of unique variants that may or may not warrant specific names to describe them. For example, in humans and mice, there are over 10 different replication–coupled H2A variants and over 10 H2B variants [6], without any clear functional distinctions among them. Faced with this challenge, attendees at the EMBO Workshop on Histone Variants that took place in October, 2011 in Strasbourg, France, found it desirable to develop some consistent rules to apply when naming variants, both to minimize confusion and to aid searching.

## Approach and rationale

Here, we begin by surveying the format and rationale of existing histone nomenclature, extracting general usage principles and noting some examples of potential conflicts arising from inconsistent use. We then propose a phylogeny-based nomenclature that utilizes consistent punctuation with letter and number suffixes and (rare) prefixes to arrive at a cogent machine-searchable scheme that is based on expectation of common structure and function through orthology, but which is flexible enough to accommodate new discoveries that will emerge from genome sequencing projects in the coming years.

We limit our discussion to the naming of histone proteins, and not the genes that encode them. Nomenclature rules for genes differ among different organisms. Some gene names may refer to the location and organization of genes or gene clusters that are unique in each species. Furthermore, multiple genes often encode an identical histone variant (for example, the *H3.3A* and *H3.3B* genes of *Drosophila* and humans, and the multiple genes encoding H3.2 and H4 in most animals), leading to ambiguity for a nomenclature basing protein names on gene names or vice versa. We, therefore, leave the naming of genes to the respective organismal research communities, and accept that histones may have organism-specific protein names based on the genes that encode them, in addition to the names discussed here based only on their amino acid sequence.

## Format of existing nomenclature for the core histones

The first core histone variants were denoted with suffixes that included numbers or letters separated by punctuation.

1. Numbers were first used to distinguish paralogs in the same organism, without knowledge of whether or not the proteins were functionally equivalent. Numbers were assigned apparently arbitrarily after chromatographic separation in the case of sea urchin H2B paralogs [3] or according to their family and their mobility in acetic acid/urea/triton X-100 (AUT) gels in the case of mammalian H2a.1, H2a.2, H2b.1, H2b.2. H3.1, H3.2, and H3.3 [7]. More recently, H3 paralogs H3.4 [8] and H3.5 [9] were added, with numbers based on order of discovery rather than electrophoretic mobility.

2. Soon after the introduction of number suffixes, variants followed by letters were introduced to indicate minor subtypes within the H2A family that had previously been overlooked: H2A.X and H2A.Z [10]. Other letter suffixes followed, with or without punctuation: H3t [11]; H3(P) [12]; H2A.Bbd (originally H2A-Bbd) [13,14]; H2BFWT [15]; H2Abd [16]; H3.X, H3.Y [17]; and so on.

3. The letter V was employed in a variety of organisms simply to indicate a difference from another sequence in the same family: H2AvD or H2Av (*Drosophila*) [18,19]; H2Bv (*Plasmodium*) [20]; H2BV, H3V and H4V (*Trypanosoma*) [21,22]. The letter B was used similarly in H3B (*Giardia*) [23].

4. Both numbers and letters have been used together as suffixes when recognized variants have paralogs: H2AL1 and H2AL2 (mouse) [24]; H2A.Z-1 and H2A.Z-2 (vertebrates) [25]; H2Abd1_c and H2Abd2_a/b (rotifers) [16]; H3v1 to H3v10 (*Stylonychia lemnae*) [26,27]; H2Asq.1 to H2Asq.3 (*Oikopleura dioica*) [28].

5. Both numbers and letters have been used for splice variants: macroH2A1.1 and macroH2A1.2 (vertebrates) [29]; H2A.Za to H2A.Zc (*Oikopleura dioica*) [28].

Some histone variants have been designated with a prefix.

1. Prefixes have marked variants that are divergent in sequence and developmentally restricted: TH2B [30] or hTSH2B [31]; gH3 [32], gcH3 [33], and leH3 [34], and so on. They have also designated variants that are structurally and/or functionally distinct: macroH2A or mH2A [35]; CenH3 [36].

2. Some variants have been assigned both a prefix and a suffix: SubH2Bv [37], soH3-1 and soH3-2 [34], and so on.

3. Specifying the histone variant of an individual species has often been accomplished with a prefix: *Hs*H2B, *Od*H4, *Pf*CenH3.

4. Often descriptors have preceded variant names, such as for variants specific to certain developmental or cell-cycle stages: for example, early H2A, cleavage stage (CS) H2B [38], replication-coupled (RC) H3 and generative cell (GC) H3s [34].

Overlaid on top of these designations has been imposed the Brno nomenclature for indicating modifications [39], which involves suffixes designating the kind and position of the modified residue and the nature of the modification, all without punctuation, for example, H3S10ph; H3K27me3; H2A.Z2K4acK11ac.

It is evident from these examples that there has been little consistency in the use of punctuation, capitalization, or prefix versus suffix to designate histone variants. There has been some consistency in the use of numbers for paralogs that have not been functionally differentiated. Some names that bear an uncomfortable resemblance to each other (for example, H2A.Bbd and H2Abd, H2Bv and H2BV) have been assigned to completely distinct variants.

## Phylogeny and historical usage

Our goal is to make guidelines that would result in logical, simple, consistent, searchable and informative names that preserve as much of the historical usage as possible. Our approach to such guidelines begins by favoring the use of information obtained from histone phylogenies to guide the creation of a logical naming system for these proteins or, at least, to be consistent with such a naming system. Indeed, the reconstruction of phylogenies has proven to be an excellent strategy in predicting structural and functional features in histones. This argument is illustrated with H2A.Z, where the detailed biochemical analysis of vertebrate-specific H2A.Z-1 and H2A.Z-2 fractions [40] was driven by previous phylogenetic analyses that pointed to their functional differentiation [25]. Therefore, a naming system based on the phylogenetic relationships among histone members within different families can help place variants in a structural, functional and evolutionary framework.

Although histones have been classically considered as archetypal examples of slow-evolving proteins, some histone variants do evolve very quickly, particularly those specifically associated with the germinal lineage (that is, H2A.Bbd, sub-acrosomal H2Bs, and so on). The presence of such heterogeneity in evolutionary rates mirrors heterogeneous selective constraints in diverse functional backgrounds, operating over the genetic diversity generated through birth-and-death during the long-term evolution of histones [25,41]. Genome sequences already tell us that there are many amplifications of variants that are particular to specific lineages [6,28,38,42,43]. Consequently, we recognize that the phylogeny and orthology of histone variants are not always clear. In a family of proteins encoded by multicopy genes, names will, therefore, commonly be used to specify a class of related orthologs and paralogs rather than a specific protein sequence (for example, H2A.Z, not *Od*H2A.Zb), and flexibility to accommodate phylogenetic uncertainty is necessary.

We also recognize that changing the names of proteins is inherently confusing and disruptive to literature searches. While some name changes are necessary to create a coherent system to guide the naming of new variants, renaming should be minimized. In some cases, compromises in naming conventions to accommodate historical usage are preferable to complete logical consistency. These considerations prevent a strictly phylogenetic approach, but encourage a flexible approach that aims to incorporate phylogeny where practical.

The guidelines we propose are described below and summarized in Table 1.

**Table 1:** Summary of nomenclature guidelines

**Naming feature**	**Recommendation**	**Examples**
Core histone name	Use in an inclusive sense for the protein family. Specify subgroups with a descriptor, prefix, letter suffix, or number suffix.	‘H2A can be ubiquitylated.’ ‘H3 can be methylated on K4.’
Capitalization	Upper and lower case are equivalent in meaning, but upper case is preferred for designating core histones, their suffixes, and modifiable amino acids. Use lowercase for modifications and for prefixes.	H3.3K4me3, H2BK123ub1, cenH3
Descriptors	Descriptors can be used before the core histone name to specify a feature, group variants developmentally or functionally, indicate the species of origin, or other uses. There should be a space between the descriptor and the core histone name. There is no requirement that a descriptor specifies a clade.	RC H2A, early H4, testis-specific H3.4 or TS H3.4, *Hs* H2A.X or human H2A.X, GC H2As, oocyte H1s
Prefixes	These should be few in number and specify a structurally distinct clade of a core histone that is universal or characteristic of a high-level taxonomic clade. Lower case is preferred for prefixes.	macroH2A, cenH3, subH2B
Letter suffixes	These should be preceded by a period (.) and specify a structurally distinct monophyletic clade of a histone family (exception: H2A.X). A suffix may be applied judiciously at any taxon level.	H2A.Z, H3.X, H2A.B
Number suffixes	These should be preceded by a period (.) and specify a particular variant of a core histone, without constraint as to distinctiveness and without implication as to phylogeny. Number suffixes should be assumed to be species-specific, but it is convenient to name variants in related species consistently where unique orthologies are clear. A number suffix should be the default designation of new variants.	H3.5, H2A.1, macroH2A.2, H1.0
Punctuation	Use a period (.) after core histone names to indicate a subtype (letter or number suffix). Use additional periods as necessary to separate finer divisions of subtypes. A period is equivalent to a branch point in a phylogenetic tree.	H2A.Z.1, H2A.L.1
Splice variants	Use a period (.) before a splice variant number. Treat the same as paralog number suffixes, except that a lowercase ‘s’ may precede the number to indicate that the isoform is a splice variant.	macroH2A.1.2, H2A.Z.s3
Synonyms	For names changed by this nomenclature, refer to both old and new synonyms in the abstract of papers to facilitate literature searches. Optional descriptors can aid identification.	‘Avian H1.0, also known as H5’

## Capitalization

From the point of view of effectively designating histone variants, we see no reason to prefer upper or lower case, or to differentiate between them. Most search engines do not distinguish upper and lower case. However, there are two reasons to prefer upper case as the default in naming variants. First, some genetic nomenclature systems differentiate proteins from genes by using uppercase for proteins. Second, the Brno nomenclature for histone modifications specifies that the protein and its modified residue(s) are upper case, while the modifications are lower case. The use of uppercase in H2A, H2B, H3, H4 and their suffixes would aid implementation of the Brno nomenclature without affecting the ability to search.

Traditionally, some histone variant prefixes (for example, macroH2A) have not been capitalized, while others have been (for example, TH2B). The use of lower case in prefixes seems unlikely to create confusion with Brno modification nomenclature or gene names, and makes the Ciba core histone family designation more visually prominent. We, therefore, prefer the use of lower case for prefixes (for example, macroH2A, subH2B, cenH3). The use of lower case should not affect searching for existing literature that uses uppercase prefixes.

## Prefixes

The majority of histone variants have been designated by suffixes, and only a handful have used prefixes. Of these prefixes, only macroH2A, cenH3, and TH2B have an extensive literature.

**Figure 1:**
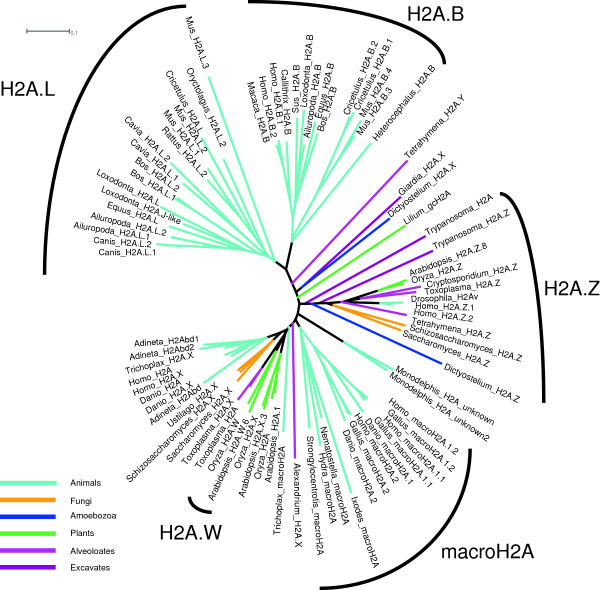
**Unrooted H2A phylogeny.** H2A.Z is a monophyletic clade present in all eukaryotes, while macroH2A (mH2A) is restricted to animals and H2A.B (H2A.Bbd) and H2A.L (H2AL) are confined to mammals. Paraphyletic or polyphyetic H2A.X and replication-coupled H2As have diverged repeatedly. Alignments and trees constructed using default ClustalW parameters and displayed using Dendroscope [45].

‘macroH2A’ or ‘mH2A’ describes a well-delimited clade of proteins containing the ‘macro-domain’ (Figure 1), which sets these proteins apart from other H2A variants [35]. macroH2A is found in diverse animal phyla, including such basal lineages as cnidarians and placozoans [44], with subtypes and splice variants in vertebrates [29]. It is likely to have been ancestrally present in animals, but has been lost in several lineages, including *Drosophila* and *Caenorhabditis*.

‘CenH3’ was proposed as a functional (rather than structural) class [36] because the monophyly of centromeric H3 proteins is uncertain [27,46,47], although monophyly seems the most parsimonious hypothesis. Either way, cenH3s form a distinct group with recognizable structural features [47,48].

**Figure 2:**
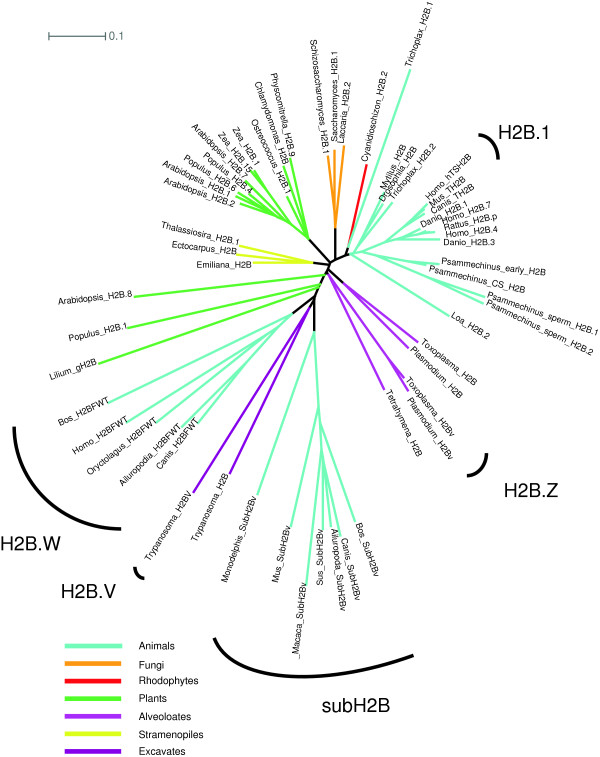
**Unrooted H2B phylogeny.** TS H2B.1 (TH2B), H2B.W (H2BFWT) and subH2B (SubH2Bv) are mammal-specific clades. Highly divergent generative cell H2Bs in plants do not form a clear clade. Apicomplexan H2Bv does not appear to be related to trypanosome H2BV, despite the fact that both are thought to interact with H2A.Z.

The variant SubH2Bv was described in bull sperm, and potential orthologs with characteristic divergent histone fold domains have been identified in mice (H2BL1) and other mammals [24,37]. This apparently rapidly evolving protein variant (Figure 2) is reportedly found in the subacrosome of primates, rodents, and marsupials as well as bovids [37,49]. The use of the prefix ‘sub’ for this family seems appropriate, since residence in the subacrosome rather than the nucleus is a distinctive property that appears to be distributed throughout mammals. We recommend dropping the superfluous suffix ‘v’. Specific recommendations for renaming variants are summarized in Table 2.

**Table 2:** Specific name change suggestions for histones

**Old name**	**Organism(s)**	**New unified name**
H2A (with SPKK motifs)	plants	H2A.W
H2A.Bbd	mammals	H2A.B
H2Abd1, H2Abd2, H2Abd	bdelloid rotifers	(bdelloid) H2A.1 to (bdelloid) H2A.3
H2AL	mammals	H2A.L
H2Av, H2AvD, D2, hv1, Htz1p	*Drosophila, Tetrahymena, Saccharomyces*	H2A.Z
SubH2Bv	mammals	subH2B
H2BL1	mammals	subH2B
H2Bv	apicomplexans	H2B.Z
H2BV	trypanosomes	H2B.V
H2BFWT	mammals	H2B.W
TH2B, hTSH2B	mammals	(TS) H2B.1
H3(P)	*Moneuplotes*	H3.P
H3t	mammals	(TS) H3.4
H3v1 to H3v10	*Stylonychia*	H3.1 to H3.10
H3V	trypanosomes	H3.V
H3.X	human	H3.Y.2
H3.Y	human	H3.Y.1
H4V	trypanosomes	H4.V
H1°	animals	H1.0
H5	birds	H1.0
H1δ	echinoderms	H1.0
H1t	mammals	(TS) H1.6
H1T2	mammals	(TS) H1.7
H1oo	mammals	(OO) H1.8
Hils1	mammals	(TS) H1.9
H1x	vertebrates	H1.10
B4	frogs	(Amphibian) H1.4

Parentheses ( ) indicate an optional descriptor.

From these examples, we suggest that prefixes be used sparingly to designate structurally distinct families of variants with wide distribution within a ‘high-level’ clade. ‘High-level’ may be taken to correspond to traditional classes, phyla, kingdoms or more inclusive clades. Of existing variants not designated with prefixes, H2A.Z would be an obvious candidate for prefix designation by our criteria if it were not the prototypical example of using letter suffixes for variants.

‘TH2B’ describes a mammalian testis-specific H2B, which can dimerize with H2AL1/L2, and may form subnucleosomal particles in condensed spermatids [24,30]. hTSH2B is the human ortholog of TH2B in rodents [31] and should be designated by the same name. Although TH2B is somewhat diverged from other vertebrate H2Bs, it falls well within the clade of animal replication –coupled H2Bs (Figure 2), and is primarily distinguished by its testis-specific expression. It is the first H2B gene encoded in the major mammalian histone gene cluster, and is designated as ‘type 1’. We, therefore, suggest that mammalian testis-specific H2B.1 (TS H2B.1) would be a better designation for this variant, where ‘H2B.1’ is the name and “mammalian testis-specific’ is a descriptive phrase that helps alert readers to its properties.

**Figure 3:**
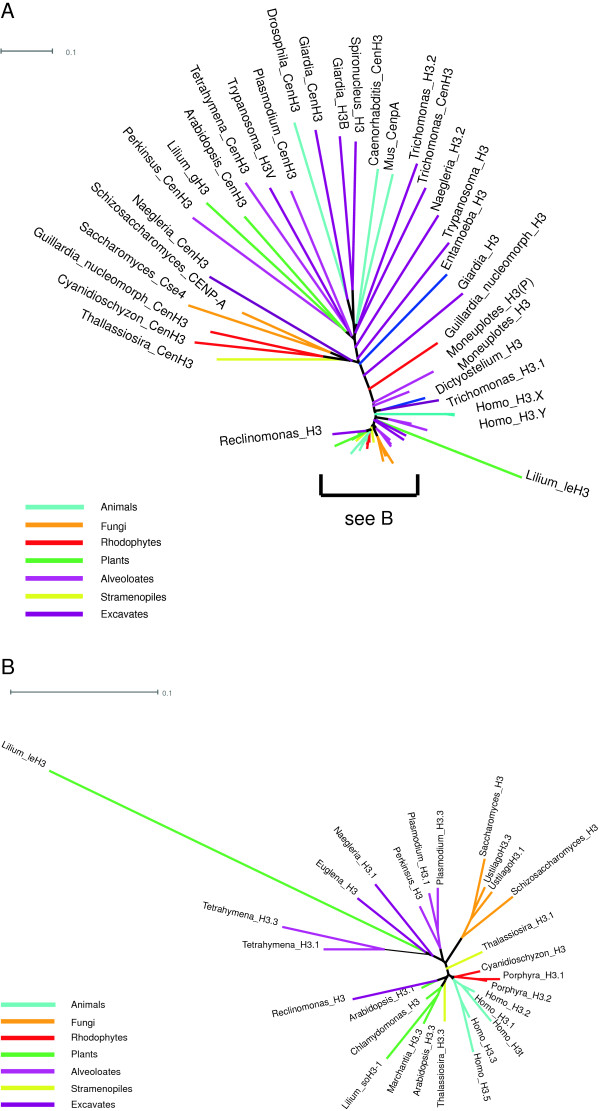
**Unrooted H3 phylogeny. (A)** cenH3s are not clearly separable from the divergent H3s of excavates and of plant generative cells. **(B)** Replication-independent H3.3s and replication-coupled H3s have diverged repeatedly in different lineages.

Other existing prefixes include those used for the five H3 variants expressed in the generative cell (GC) of *Lilium*[34]*.* Of these, gcH3 and leH3 have deletions in the histone fold domain and are probably non-functional, although they might have non-nucleosomal functions like SubH2Bv. gH3 makes a chromatin protein, but there is no evidence at present that it is not a *Lilium*-specific variant (Figure 3A). soH3-1 and soH3-2 are subtypes of H3.3. All five of these variants should be designated by paralog numbers until such time as they are demonstrated to represent widespread subtypes.

## Descriptors

The continued use of the descriptive phrase ‘GC H3 variants’ for these *Lilium* H3s [34] and for similar variants in *Arabidopsis* and other plants would be consistent with our proposal. In this case, ‘GC’ is not a prefix, but is separated by a space from the name, and acts as a descriptor to specify that the variants are found in generative cells. Such descriptors may be applied to describe functional, stage-specific or other groupings of variants that do not necessarily form phylogenetic clades. The (roughly) corresponding descriptor for animals is testis-specific (TS). TS variants appear to be widespread, rapidly evolving and polyphyletic in animals [9,28,38]. The same seems likely to be true of GC variants in plants. We encourage the use of flexible, detached descriptors rather than permanent prefixes or suffixes to specify developmental stages or cell-type specific expression of histone variants, because subsequent work may reveal a wider developmental deployment for a variant than was initially discovered. We specifically encourage the use of descriptors, such as TS, to specify testis-specific histones, including TS H2B.1 in mammals and the non-orthologous TS H2Bs found in other groups of animals [28,38].

Descriptors may also be used to designate the organism. As with other descriptors, a space should be maintained before the variant name to facilitate machine searches and reduce possible confusion (*Hs* H2A.Z or human H2A.Z, not HsH2A.Z). Under our scheme, the three classes of bdelloid rotifer H2Abd, which all have long but apparently unrelated tails, might be better designated with a species or group descriptor and paralog numbers (*Adineta vaga* H2A.1 to H2A.3 or bdelloid H2A.1 to H2A.3) since it is presently unclear whether these variants form a clade or are polyphyletic (Figure 1). If it can be shown that they form a clade, then a common prefix or letter suffix would be appropriate.

While descriptors are intended to be flexible and unregulated, we suggest that a practical order for the use of multiple descriptors might be (organism or group) (developmental stage and/or tissue) (other) before the Ciba designation and any suffix. Descriptors are optional, but are encouraged where they are informative, especially upon first occurrence of a variant name in a manuscript.

## Suffixes

Letter suffixes function essentially the same as prefixes in designating structurally distinct variants, except that they need not characterize a high-level clade. We prefer the use of single letter suffixes preceded by a period, as in the prototypes H2A.X and H2A.Z. Thus H2A.W is proposed for the plant-specific clade of H2As (Figure 1) with putative minor-groove-binding motifs (SPKK) in their tails that wrap more DNA than other H2As [50]. We further suggest H3.P for *Moneuplotes* H3(P), H2A.B for mammalian H2A.Bbd, and H2B.W for mammalian H2BFWT. The continued use of ‘H2A.Bbd’ would still be searchable with ‘H2A.B*’, so in this case the traditional name could still be used by those who prefer it, though we suggest transitioning to ‘H2A.B’. ‘H2BFWT:’ has only two PubMed hits [15,51], and NCBI already refers to this family as ‘member W’ or ‘type W-T’, so we think it would be minimally disruptive to change its designation to ‘H2B.W’. Although it may eventually be necessary to use multiple-letter suffixes, we prefer to stick with single letters until they become insufficient.

H3.X and H3.Y are part of a primate-specific clade that is distinct from other H3s, but they are highly similar to each other; so far, the *H3.X* gene has not been shown to express protein [17]. We suggest designating these variants H3.Y.1 and H3.Y.2 to better reflect their close relationship, and to save ‘H3.X’ for a verified protein with a distinct structure. In general, in order to avoid running out of unique letter suffixes, we suggest that the default for designating new variants should be the assignment of paralog numbers, with the assignment of letters judiciously applied to variants with obviously unique features. For example, the H2A.Y variant from *Tetrahymena* clearly warrants a letter suffix because of its unique leucine-rich-repeat domain and phosphatase regulating activity [52]. In contrast, two human H2A.Bbd variants differ by only a single amino acid of unknown significance and should be distinguished through paralog numbers: H2A.B.1 and H2A.B.2.

We advocate the continued use of paralog numbers preceded by a period to designate individual variants of one family without implication as to their phylogenetic interrelationships or functions. Paralog numbers should be assumed to be organism-specific unless otherwise indicated. Thus H2A.1 of humans would not be assumed to be the same as H2A.1 of *Arabidopsis* or of *Oikopleura*. However, the assignment of paralog numbers in one organism should be consistent with paralog numbers in related organisms if unique orthologies are clear. For example, vertebrates have two macroH2A proteins, one designated macroH2A1 or macroH2A.1 that is orthologous to the macroH2A.1 originally described from rat liver [35], and one designated macroH2A2 that is orthologous to the macroH2A2 first described from humans [53]. There is no need to assign paralog numbers sequentially if there is some phylogenetic, mnemonic or other reason to prefer a non-sequential number, such as correspondence to the names of orthologs, pre-existing gene numbers, or to names assigned by gene organization.

The (non-centromeric) H3 variants constitute a special case because they are few in number and have the possibility of a fairly complete phylogeny [27,54-57], and also because of the historical usage of the human paralog numbers H3.1, H3.2 and H3.3. Phylogenetic analysis has inferred that both replication-coupled (RC) H3s and replacement or replication-independent (RI) H3s have most likely arisen polyphyletically (Figure 3B). Current usage often distinguishes between ‘H3’ for RC forms and ‘H3.3’ for replacement forms in a variety of organisms [7,58-62]. In contrast, ascomycetes generally have only a single form which is H3.3-like but is usually referred to simply as ‘H3’ [63]. Unqualified ‘H3’ is also used in an inclusive sense for all H3 variants in the many contexts in which the variants cannot be distinguished. We encourage the use of this inclusive meaning for ‘H3’ (without descriptors), which would, therefore, continue to apply to ascomycetes, and the use of an organism-appropriate paralog name (H3.1, H3.2, and so on.) or descriptors such as ‘RC’ to indicate replication-coupled H3 variants. H3.1 is a more recent mammal-specific divergence from H3.2, which is the RC H3 variant found throughout animals [27].

Following the usage in animals, ‘H3.3’ has been applied in plants [42,58] and alveolates [57,60,61] to indicate RI variants. Given the likelihood of independent divergences, these variants are neither more nor less orthologous to animal H3.3 than their RC counterparts, but it would be highly inconvenient to alter this practice. This highlights a useful feature of paralog numbers: since paralog numbers are not intended to imply unique corresponding orthology across organismal groups, the use of ‘H3.3’ in multiple kingdoms does not misrepresent orthology, but functions as a well-established way of indicating RI variants in a variety of organisms that is shorter than using a descriptor, such as ‘replacement’ or ‘RI’ H3. RI and RC variants within an organism typically differ in residue 31 (and whether it can be phosphorylated) as well as residues 86 and 89, as shown in Table 3, but distinguishing residues vary in different organisms and caution is advised in designating ‘H3.3’ in less well-studied eukaryotic kingdoms.

**Table 3:** Sequence variation in ‘H3.3’ and ‘H2A.X’ variants

Kingdom	Organism	Histone Variant	Residue 31 -	Residues (85)86-89
Animals	*Homo*	H3.3	**S**	**A**A**IG**
	*Homo*	H3.2	A	SAVM
Fungi	*Saccharomyces*	H3	S	SAIG
Plants	*Arabidopsis*	H3.3	**T**	**H**AV**L**
	*Arabidopsis*	H3.1	A	SAVA
Rhodophytes	*Porphyra*	H3.3?	**S-**	**T**AVL
	*Porphyra*	H3.1?	VG	SAVL
Alveolates	*Tetrahymena*	H3.3	**VS**	**Q**A**I**L
	*Tetrahymena*	H3.1	AT	SAVL
Heterokonts	*Thalassiosira*	H3.3?	**TA**	**ST**AVL
	*Thalassiosira*	H3.1?	AT	GSAVL
Amoebozoa	*Dictyostelium*	H3.3?	**STQP**	AAI**Q**
	*Dictyostelium*	H3.1?	VNEV	AAIE
Excavates	*Euglena*	H3	A	NAIL
			**Residues** ** 109-**	
Animals	*Homo*	H2A.X	PNIQAVLLPKKSATVGPKAPSGGKKATQA**SQEY**	
	*Homo*	H2A.2.2	PNIQAVLLPKKTSHKPGKNK	
Fungi	*Saccharomyces*	H2A.X	PNIHQNLLPKKSAKATKA**SQEL**	
Plants	*Arabidopsis*	H2A.X.3	PNIHQTLLPSKVGKNKGDIGSA**SQEF**	
	*Arabidopsis*	H2A.1	PNIHNLLLPKKAGASKPQED	
Rhodophytes	*Griffithsia*	H2A.X	PNIHQVLMPRKKTKGDA**SQEV**	
	*Cyanidioschyzon*	H2A	PNIHAVLLPKKKAKGE	
Alveolates	*Tetrahymena*	H2A.X	PNINPMLLPSKSKKTESRGGA**SQDL**	
	*Tetrahymena*	H2A.1	PNINPMLLPSKTKKSTEPEH	
Heterokonts	*Phaeodactylum*	H2A.X	PNIHAILLPKKTIKTKGP**SQDY**	
	*Phaeodactylum*	H2A.3	PNIHAILLPKKSGPTK	
Amoebozoa	*Dictyostelium*	H2A.X?	PTPQQSTGEKKKKPSKKAAEGS**SQIY**	
	*Dictyostelium*	H2A	PTPQSNTEGKKKKATSKKS	
Excavates	*Giardia*	H2A.X	RSAKEGREGKGSHR**SQDL**	
	*Trypanosoma*	H2A	PSLNKALAKKQKSGKHAKATPSV	

RI ‘H3.3’ variants and ‘H2A.X’ variants are paraphyletic or polyphyletic, but have recognizable sequence features. Upper panel: Divergence between RI and RC H3 variants usually involves differences at residue 31 and residues 86 to 89. Lower panel: ‘H2A.X’ variants are distinguished from related H2A variants by bearing a consensus SQ(E/D)Φ phosphorylation motif at the C-terminus. Residue numbers refer to the human H3.3 and H2A.X protein sequences, and to orthologous positions in other variants.

## Punctuation

Punctuation and no punctuation are both currently used when appending sub-designations to basic histone families. Punctuation is convenient for separating numeric paralog designations from the alphanumeric Ciba names of the histone families (for example, H3.3, not H33). Periods, dashes, and parentheses have been used without any distinction in meaning, but the period is the most common form of punctuation in histone variant names, and is essential for finding the relevant literature on these variants in PubMed. In addition, the use of other special characters, such as parentheses, dashes, slashes, superscripts and subscripts, and so on, can complicate searches. We suggest the use of a period to separate each appended sub-designation (except unpunctuated modification designations, in keeping with the Brno nomenclature).

In both letter and number suffixes, the period functions essentially to designate a branchpoint in a phylogenetic tree: H2A.Z, H2A.Y and H2A.1 represent different branches of the H2A family. The use of the original name ‘macroH2A.1’ for vertebrate macroH2A1 [35] would extend this principle to the vertebrate branches of the macroH2A family, if the corresponding form ‘macroH2A.2’ were used for the original form ‘macroH2A2’ [53].

This logic of designating branchpoints with a period can be extended to subsequent branchpoints as needed. Thus, vertebrate H2A.Z-1 and H2A.Z-2 can be represented as H2A.Z.1 and H2A.Z.2, indicating the two branches of the H2A.Z subfamily. Similarly, mouse H2AL1 and H2AL2 can be designated H2A.L.1 and H2A.L.2, and *Oikopleura* H2Asq.1 to H2Asq.3 can be H2A.Q.1 to H2A.Q.3 or similar designation.

Although paralog number suffixes are not generally intended to mark clades, in some cases variants that are not distinctive enough to warrant a letter suffix, nevertheless fall into recognizable clades with subtypes that can be described using a period and additional suffix. For example, human H2A.1 and H2A.2, as originally defined electrophoretically, actually represent two subfamilies of H2A variants that differ by whether they have leucine or methionine at position 51. By designating the individual variants in these subfamilies using an additional branchpoint (for example, H2A.1.6 or H2A.2.3), individual variants can be uniquely designated while retaining H2A.1 and H2A.2 for the original subfamilies as defined electrophoretically.

The same logic could be applied to any case when an organism has multiple similar variants that group into subfamilies, but in some cases such phylogenetic detail may be more distracting than informative. Mammalian H3.1 is clearly derived from animal H3.2, but there seems little advantage in designating mammalian H3.1 and H3.2 as ‘H3.2.1’ and ‘H3.2.2’, although both pairs of designations would be allowable under our nomenclature guidelines. Similarly, where there is more than one H3.3-like variant in an organism, as is the case for *Caenorhabditis* and many plants [64], it will usually be simplest to assign different paralog numbers to the individual variants, presumably with ‘H3.3’ assigned to the most abundant or appropriate such variant. For example, in *Arabidopsis*, the germline-specific RI variant known as HTR10 (a gene-derived name) or AtMGH3 (a prefix of the type we discourage here) [42,65] might be designated ‘H3.10’ to distinguish it from the ubiquitous RI variant ‘H3.3’, while avoiding the equally correct but more cumbersome designation ‘H3.3.10’. In general, we believe that multiple numeric suffixes can become confusing and that shorter names are preferable, unless there is a compelling reason to provide a name that incorporates detailed phylogeny. In names, as in phylogenetic trees, clarity is usually more important than representing every known branchpoint.

We see no reason to treat splice variants differently than paralogs, so the same branchpoint logic can be usefully applied to splice variants: macroH2A1.1 and macroH2A1.2 can be designated as macroH2A.1.1 and macroH2A.1.2, while *Oikopleura* H2A.Za to H2A.Zc [28] would become H2A.Z.1 to H2A.Z.3. While the latter might lead to confusion with vertebrate H2A.Z.1 and H2A.Z.2, this ambiguity is inherent in the use of organism-specific paralog numbers, which seems unavoidable given the ubiquity of lineage-specific expansions of variants. When it is desirable to distinguish splice variants from paralogs for clarity, we suggest allowing the use of the lowercase letter ‘s’ (for ‘splice variant’) before the splice variant number, for example macroH2A.1.s1 or H2A.Z.s2.

The imposition of this formal punctuation is intended to apply to written designations, not to impose a stilted formality to speech. In common usage, (.) is pronounced “point” before numbers (for example, “ π = 3.14” or “ histone H3.3”) and “dot” before letters (for example, “NIH.gov”). The “dot” is often dropped in pronouncing variants like H2A.Z or H2A.X. We have no intention of interfering with these or other patterns of natural speech. We only seek consistent punctuation in written names to achieve uniform spelling rules that aid searching and express phylogenetic relationships.

## Synonyms and homographs

The use of alternative names for the same or the orthologous variant should be discouraged, except to list synonyms. Thus, we favor using H2A.Z in preference to *Saccharomyces* Htz1p, *Tetrahymena* hv1, or *Drosophila* H2Av/H2AvD/D2. H2Av has a convergent phosphorylation motif that allows this protein to function similarly to H2A.X. This does not alter the fact that this protein is a legitimate H2A.Z, but it suggests that the alternative name H2A.Z.X might be useful for discussing this protein in contexts that concern its phosphorylation. The phosphorylated state can either be denoted according to the Brno nomenclature (H2A.ZS138ph) or as γH2A.Z.X, in parallel with the usual γH2A.X.

The cases of CENP-A [66], Cse4p [67] and some other centromeric H3s are somewhat exceptional in that these names are earlier and have priority over cenH3, and are well-established in animal and yeast literature. The established use of multiple names was part of the rationale for creating a functional category to apply to all centromeric H3s regardless of monophyly. PubMed treats CENP-A and cenH3 as synonyms, and Cse4p is well-known to centromere researchers, so the (im)practical consequences of synonymy are largely ameliorated. Nevertheless we encourage the use of cenH3, especially when the context is chromatin or histones, and in organisms in which orthology to animal CENP-A or fungal Cse4 is uncertain.

**Figure 4:**
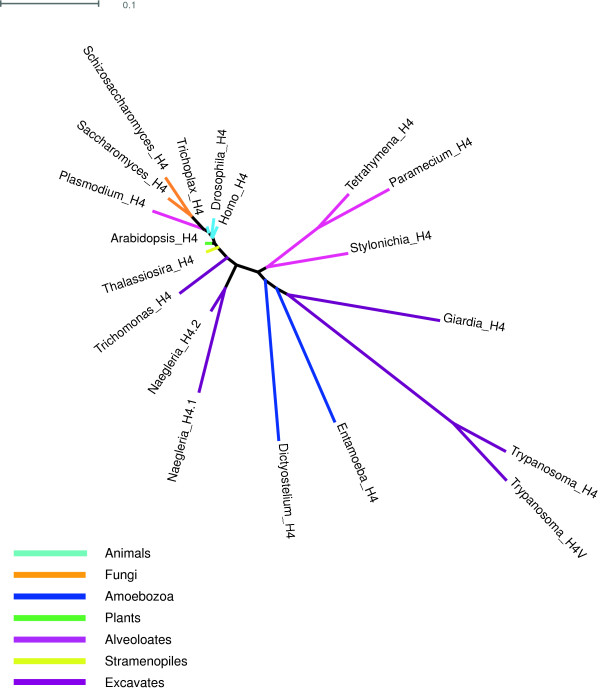
**Unrooted H4 phylogeny.** Most eukaryotes have a single form of H4, and most divergence in H4s is found in excavates, amoebozoans, and ciliates versus other eukaryotes.

H3t has now been applied to both humans and urochordates, but the proteins are not orthologous [28]. We recommend using the descriptor TS before these: TS H3.4 (the original name of human H3t) [8] in humans and TS H3.4.1 to H3.4.3 in urochordates (no orthology with human H3.4 implied) [28]. H2Bv (or H2BV) has been used in *Plasmodium, Toxoplasma* and *Trypanosoma*[20-22]. In *Plasmodium* and *Toxoplasma*, the two H2Bvs are apparent orthologs, but this is unlikely to be the case for the *Trypanosoma* variant (Figure 2). Priority for the name H2BV goes to *Trypanosoma*[21], which also has H3V and H4V [22]. We suggest these be reformatted to H2B.V, H3.V, and H4.V (Figure 4). In *Toxoplasma*, H2Bv is associated with H2A.Z [68] (which intriguingly is also true in *Trypanosoma* H2BV), suggesting that an alternative name that does not imply identity with the *Trypanosoma* variant might be H2B.Z. H3v has been used with paralog numbers (H3v1 to H3v10) for the many H3 variants in *Stylonychia*[26,27]. We suggest that the ‘v’ in these names be replaced with a period (H3.1 to H3.10), in keeping with other paralog number designations.

## Additional considerations

As with ‘H3’, we encourage the use of ‘H2A’ and ‘H2B’ in the inclusive sense, and the use of a descriptor, such as ‘RC’, to specify replication-coupled forms. ‘H2A.X’ has traditionally been used to designate the subset of H2A variants that bear a terminal SQ(E/D)Φ phosphorylation motif, often used in contrast to unqualified “H2A”, as shown in Table 3. Phylogenetic analysis indicates that SQ(E/D)Φ−bearing variants have diverged repeatedly from variants lacking this motif [47]. This makes the designation of SQ(E/D)Φ−bearing variants as ‘H2A.X’ either paraphyletic or polyphyletic, depending on whether the motif is ancestral or not. Despite our desire to use letter suffixes for monophyletic clades, we see no easy solution to this other than to continue with historical usage. The situation is analogous to that with H3.3, except that we allow number suffixes to be organism-specific without phylogenetic implication.

H2A.Bbd [13] (or as we would prefer, H2A.B) is a growing subfamily of histones that appears to be related to the H2AL (or H2A.L) subfamily [24]. Both families are involved in mammalian spermiogenesis [24,69,70], and are rapidly evolving, with lineage specific expansions (Figure 1). Both have shortened docking domains and wrap less DNA than other H2As [14,71]. H2A.L forms subnucleosomal particles with TH2B (TS H2B.1) [24]. When these two H2A subfamilies are better understood, there may be a logical method of combining them with a prefix for short wrapping (or another characteristic); however, they represent distinct clades, and both are widely distributed in mammals, so we conservatively recommend treating them as distinct variants at the present.

In general, many new variants are likely to be testis-specific or pollen-specific, and we urge caution and conservatism in assigning them new names. Indications are that these variants are common, polyphyletic, rapidly evolving and may have unusual properties. Orthologies and paralogies may be difficult to disentangle. We recommend naming variants initially with paralog numbers and then renaming them when their properties and relationships are better understood. We encourage the use of descriptors (for example, TS) rather than prefixes and letter suffixes when only tissue- or cell-specific expression patterns distinguish these variants from other similar variants.

## Nomenclature for histone H1

Histone H1 differs dramatically from the core histones. It has an entirely separate origin, probably from bacterial proteins rather than from archaeal histones [72]. Rather than a histone fold, H1s typically have a short basic amino-terminal domain, a globular winged-helix domain and a lysine-rich carboxy-terminal domain often characterized by a proline-kinked alanine-lysine helix. H1s are less conserved than other histones. In some unicellular eukaryotes, such as Euglenozoa and Alveolata, H1s lack the winged-helix domain.

Most studies of H1 have taken place in animals. The discovery of H1 variants in calf thymus preceded the discovery of core histone variants by several years [73,74], and 11 variants have now been identified in humans [75,76]. As with core histones, a variety of naming styles have been applied to H1s in different organisms, including paralog numbers, letter suffixes, and combination letter and number suffixes. An early and widely-used nomenclature used lower case letters to designate paralogs in the order of elution from a Bio-Rex 70 column [77], and was subsequently adopted for variants separated on 2-D gels [78,79]. Confusion over some 12 different nomenclatures led to a previous attempt to create a system in which variant designations were applied uniformly to orthologs across mammalian species [80].

The cloning of human H1 genes introduced a nomenclature that more closely resembles core histone names in the use of a period before a paralog number [81-83], and that is now commonly used for human variants. Human H1s are often subdivided by the use of descriptors into somatic H1s and germ cell H1s. The somatic H1s include H1.1 to H1.5, H1x, and H1° or H1.0. Germ cell H1s have been designated H1t, H1T2, Hils1 (all testis-specific) and H1oo (oocyte-specific). H1s in other less well-characterized organisms are designated with paralog numbers, lower case letters, or even Greek letters. Can the phylogenetic approach and conventions proposed here for the core histones be of help for standardizing H1 nomenclature?

**Figure 5:**
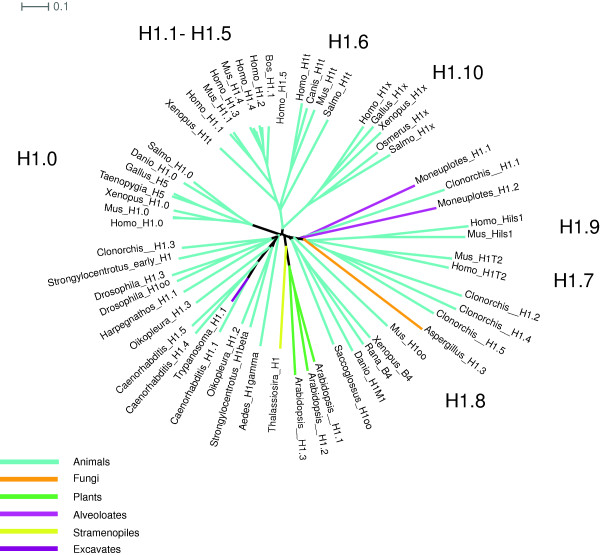
**Unrooted H1 phylogeny.** H1.0 (H1°) is an animal-specific clade, including avian H5. H1.10 (H1x) is found in vertebrates. Mammal-specific clades include H1.7 (H1T2) and H1.9 (Hils1). H1.8 (H1oo) and H1.6 (H1t) are also monophyletic in mammals, but other TS H1s and oocyte H1s are not clearly members of the same clades.

Constructing a phylogeny of H1s yields a ‘star’ pattern with long branches converging on a center that has low resolution of branching (Figure 5). The short branches in the center are unstable with respect to the choice of the substitution matrix used to construct the tree. Several animal-specific lineages appear to be as distantly related to each other as they are to lineages in other eukaryotes. While the underrepresentation of most eukaryotic groups contributes to the poorly resolved branching, the larger factor is likely to be the relatively faster rate of evolution for H1s, especially germ cell H1s, over core histones, leaving distant homologs too diverged to construct an informative tree. The sheer number of H1 variants places constraints on naming them, because with 11 variants just in humans, it is easy to foresee running out of single letters to use as suffixes. Thus the default for discovering new H1s should be to assign them organism-specific paralog numbers, since distant orthologies that would support a letter suffix are unlikely to be verifiable.

Mammals present some exceptions to this lack of detectable orthology. The human H1 variants H1.1 to H1.5 form a clade, and the individual variants have orthologs in other mammalian species, which can be clearly identified by their gene organization as well as their sequence [6]. However, these orthologs in different species have not always used the same nomenclature (for example, human H1.4 vs. mouse H1e), nor been assigned the same paralog numbers (for example, bovine H1.1 is not orthologous to human H1.1). The consistent use of the same paralog numbers for orthologs in different mammals has been the goal of a unified nomenclature for over 15 years, and should be adopted. The designations based on cloned human genes use the same format as the core histones, and their adoption for orthologs in other mammals offers the possibility of a unifying nomenclature for all histones.

H1° is widespread in animals [84-88], and already has an alternate name, H1.0, that conforms to our proposed nomenclature. H5 from chicken erythrocytes was known at its discovery to be an equivalent of H1 [89,90], and has long been known to be a specific ortholog of H1.0 [84,90,91]. Despite over 35 years of literature using ‘H5’, we find this name to be actively misinformative, since H5 does not form a separate high-level structural class of histones, and we recommend that it be replaced with ‘H1.0’, mentioning H5 as a synonym. A descriptor such as ‘avian erythrocyte’ can be added where necessary. The same nomenclature should also be applied in the case of H1.0 orthologs identified in other non-vertebrate metazoans, including the histone H1∂ from sea urchin [92] as well as RI H1 histones from bivalve molluscs [86-88]. Molecular phylogenetic analyses have revealed that these variants share a common monophyletic origin that can be traced back before the differentiation between protostomes and deuterostomes, very early in metazoan evolution.

H1x is found throughout vertebrates, but clear orthologs of the human germ cell variants H1t, H1T2, Hils1 and H1oo are restricted to mammals. Considering the possibility that every non-mammalian genome sequenced will introduce a similar number of H1s, we recommend assigning all of the H1 variants paralog numbers and doing away with letter suffixes for H1s. Table 4 lists the suggested designations for human H1s and their mammalian orthologs. The use of descriptors (for example, TS and OO) with the germ cell variants and listing of synonyms can help to ease the transition to the new nomenclature. H1x is designated by H1.10, which is intended as a convenient mnemonic for those familiar with Roman numerals. H1oo has been previously claimed to be specifically related to amphibian B4/H1M and cleavage stage H1 of sea urchins [95], but their orthology appears uncertain (Figure 5). Thus, we conservatively suggest that B4 become H1.4 of *Xenopus* (no implied orthology to mammalian H1.4) rather than assuming orthology to H1oo. The use of descriptors ‘oocyte’ or ‘OO’, ‘maternal’, ‘embryonic’, ‘cleavage stage’ or other can be used to speak collectively of functionally similar histones in diverse animals without implying orthology.

**Table 4:** Unified nomenclature and synonyms for mammalian H1 variants

			**Histone Gene Cluster 1**						
**Human**			**Other Mammals**				
**Gene symbol**	**Albig and Doenecke**[83]	**Ohe and Iwai**[93]	**mouse gene symbol**	**Parsegian and Hamkalo**[94]	**Seyedin and Kistler, Lennox and Cohen**[77,78]	**New unified**	
*HIST1H1A*	H1.1		*Hist1h1a*	H1a	H1a	H1.1	
*HIST1H1B*	H1.5	H1a	*Hist1h1b*	H1^s^-3	H1b	H1.5	
*HIST1H1C*	H1.2	H1d	*Hist1h1c*	H1^s^-1	H1c	H1.2	
*HIST1H1D*	H1.3	H1c	*Hist1h1d*	H1^s^-2	H1d	H1.3	
*HIST1H1E*	H1.4	H1b	*Hist1h1e*	H1^s^-4	H1e	H1.4	
*HIST1H1T*	H1t		*Hist1h1t*		H1t	(TS) H1.6	
**Orphan Genes**							
**Human**				**Mouse**		**New unified**	
**gene symbol**	**alias**	**full name**		**gene symbol**	**alias**		
*H1F0*	H1.0, H1°	H1 histone family, member 0		*H1f0*	H1(0)	H1.0	
*H1FNT*	H1T2	H1 histone family, member N, testis-specific		*H1fnt*	H1t2	(TS) H1.7	
*H1FOO*	H1oo	H1 histone family, member O, oocyte-specific		*H1foo*	H1oo	(OO) H1.8	
*HILS1*		Histone H1-like protein in spermatids 1		*Hils1*	TISP64	(TS) H1.9	
*H1FX*	H1x	H1 histone family, member X		*H1fx*	H1X	H1.10	

## Conclusions

We describe here a unified nomenclature for histones that is readily machine-searchable and uses a single standardized form of punctuation to delimit variants. This nomenclature encourages the use of the histone names H1, H2A, H2B, H3 and H4 to represent histone families, and the specification of particular variants within those families by the use of suffixes and a few prefixes. The variant designations incorporate phylogenetic information by attempting to restrict the use of prefixes and letter suffixes to represent monophyletic clades, with the exception of H2A.X and possibly cenH3, which designations are defined by established usage for highly conserved and clearly demarcated functions. For simplicity, we encourage the use of single letter suffixes, and recommend capitalization to be consistent with the Brno nomenclature for modifications.

This nomenclature system allows flexibility and agnosticism with regard to phylogeny of variants through the long-established use of paralog number suffixes to indicate individual unique variants on an organism-specific basis without implying phylogenetic relationships. Where orthologies are clear between related numbered variants, as in mammalian H1s, we encourage the adoption of paralog numbers that are consistent with known orthologs in related organisms, though paralog numbers should not be assumed to be orthologous between species without specific knowledge. The assignment of paralog numbers within an organism need not be sequential if a mnemonic or other purpose is served by choosing otherwise, for example, to bring gene and protein numbers into conformity. Consistent with current usage, we suggest reserving the designation H3.3 for the major replication-independent or replacement H3 in a particular organism. The system is adaptable to include information on multiple steps of phylogenetic divergence through treating each period (.) as a phylogenetic branchpoint, as in vertebrate H2A.Z.1 and H2A.Z.2. Splice variants should be treated like other paralogs, but can optionally be indicated by the addition of ‘s’ before a paralog number where desirable for clarity, as in macroH2A.1.s1 and macroH2A.1.s2.

Our system has attempted to accommodate historical usage where it does not conflict with the underlying principles and, in a few cases, where it does conflict. Where existing names are changed by our guidelines, we strongly recommend that authors include both the old and new names in the abstract of their reports to facilitate literature searches. We encourage the use of descriptors for specifying species, functional properties and tissue- or stage-specific expression. Such descriptors are intentionally not standardized to assure flexibility, though some descriptors, such as testis-specific (TS), may become commonly used. We recommend that each new histone variant by default be assigned a paralog number, with a letter suffix assigned only if helpful to call out distinctive families of variants as phylogeny and protein properties become clear. The use of new prefixes should meet an even higher standard of need and significance. An example of how to apply these guidelines to a particular organism is given in Table 5 for *Arabidopsis*.

**Table 5:** **Application of nomenclature to*Arabidopsis***

**H1**	**Gene**	**Protein**	**Former name**
At1g06760	*HON1*	H1.1	H1
At2g30620	*HON2*	H1.2	"
At2g18050	*HON3*	H1.3	"
**H2A**	**Gene**	**Protein**	**Former name**
At5g54640	*HTA1*	H2A.1	Canonical H2A
At4g27230	*HTA2*	H2A.2	"
At1g51060	*HTA10*	H2A.10	"
At3g20670	*HTA13*	H2A.13	"
At1g54690	*HTA3*	H2A.X.3	H2A.X
At1g08880	*HTA5*	H2A.X.5	"
At5g59870	*HTA6*	H2A.W.6	SPKK-bearing H2As
At5g27670	*HTA7*	H2A.W.7	"
At5g02560	*HTA12*	H2A.W.12	"
At2g38810	*HTA8*	H2A.Z.8	H2A.Z
At1g52740	*HTA9*	H2A.Z.9	"
At3g54560	*HTA11*	H2A.Z.11	"
At4g13570	*HTA4*	H2A.Z.4	none
**H2B**	**Gene**	**Protein**	**Former name**
At1g07790	*HTB1*	H2B.1	H2B
At5g22880	*HTB2*	H2B.2	"
At2g28720	*HTB3*	H2B.3	"
At5g59910	*HTB4*	H2B.4	"
At2g37470	*HTB5*	H2B.5	"
At3g53650	*HTB6*	H2B.6	"
At3g09480	*HTB7*	H2B.7	"
At1g08170	*HTB8*	H2B.8	"
At3g45980	*HTB9*	H2B.9	"
At5g02570	*HTB10*	H2B.10	"
At3g46030	*HTB11*	H2B.11	"
**H3**	**Gene**	**Protein**	**Former name**
At5g65360	*HTR1*	H3.1	H3.1
At1g09200	*HTR2*	"	"
At3g27360	*HTR3*	"	"
At5g10400	*HTR9*	"	"
At5g10390	*HTR13*	"	"
At4g40030	*HTR4*	H3.3	H3.3
At4g40040	*HTR5*	"	"
At5g10980	*HTR8*	"	"
At1g13370	*HTR6*	H3.6	none
At1g19890	*HTR10*	H3.10	MGH3/HTR10
At1g75600	*HTR14*	H3.14	none
At1g01370	*HTR12*	cenH3	CENH3/CENP-A/HTR12
At1g75610	*HTR7*	H3.7	
**H4**	***Gene***	**Protein**	**Former name**
At3g46320	*HF01*	H4	H4
At5g59690	*HF02*	"	"
At2g28740	*HF03*	"	"
At1g07820	*HF04*	"	"
At3g53730	*HF05*	"	"
At5g59970	*HF06*	"	"
At3g45930	*HF07*	"	"
At1g07660	*HF08*	"	"

## Abbreviations

CS, Cleavage stage; GC, Generative cell-specific; OO, Oocyte-specific; RC, Replication-coupled; RI, Replication-independent; TS, Testis-specific.

## Competing interests

The authors declare no competing interests.

## Authors’ contributions

PBT and SH designed the study. FB, SHo, HW and RY contributed the *Arabidopsis* tables, and RS contributed the H1 table. All other authors provided input during early discussions and drafts, and PBT wrote the paper. All authors read and approved the final manuscript.
